# NLR Is Associated With Geriatric Depression in Chinese Women: A Community-Based Cross-Sectional Study in Eastern China

**DOI:** 10.3389/fpsyg.2019.02941

**Published:** 2020-01-10

**Authors:** Meng Liang, Bingying Du, Hailing Zhang, Xiaoyan Lu, Chao Chen, Cunxiu Fan, Xiaoying Bi

**Affiliations:** Department of Neurology, Changhai Hospital, Second Military Medical University, Shanghai, China

**Keywords:** depression, neutrophil to lymphocyte ratio, inflammation, female, Chinese

## Abstract

In recent years, the prevalence of depression has been increasing, causing a serious global burden of disease. Previous studies have proved that low-grade inflammation is involved in the process of depression. To explore the role of inflammation in the pathogenesis of senile depression, neutrophil to lymphocyte ratio (NLR) was used in this study, as a biological marker for non-specific inflammation. The present study was conducted among elderly adults from a randomly selected Community in Yangpu District, Shanghai from November 1 to December 30, 2017. 665 participants were included in the present study, including 276 males (median age was 68 years old) and 389 females (median age was 67 years old). NLR was calculated based on results of hematology examination. Depressive symptoms were assessed using the simplified version of Geriatric Depression Scale (GDS), the participant with total score greater than five points was considered to be depressed. The association between NLR and depression was evaluated separately for men and women. The prevalence of depression was 35.1% for men and 34.4% for women. No associations were found between NLR and depression among males. Nevertheless, compared with normal women, NLR was significantly increased in women with depressive symptoms (*P* = 0.020). Logistic regression analysis showed that NLR was independently associated with geriatric depression in women. The odds ratios (ORs) of depression was 2.152 (1.095, 4.227; *P* = 0.026) for the fourth compared with the first quartile of NLR. In conclusion, this study showed increased NLR was significantly associated with geriatric depression in women, but not men.

## Introduction

Depression is a common mental illness with an increasing prevalence worldwide and makes an great contribution to global burden of disease (Compton et al., 2006; Ferrari et al., 2013). Depression can not only reduce patients’ quality of life, but also affect their cognitive function (Hammar and Ardal, 2009; Steger and Kashdan, 2009). What’s worse, it can increase the incidence of suicide (Miret et al., 2013). Therefore, early diagnosis and intervention for depression have important social significance.

Although the pathogenesis of depression is still not fully understood, previous studies have indicated that inflammation plays an important role in the development of depression (Patel, 2013; Kohler et al., 2016). At the same time, multiple studies have shown that antidepressants may exert their antidepressant effects through anti-inflammatory effects, which confirmed the inflammatory profile of depression (Hannestad et al., 2011). The neutrophil to lymphocyte ratio (NLR), a common biomarker for systemic inflammatory state, has been proved to be associated with many chronic diseases (Imtiaz et al., 2012), has been proved to be related with depressive disorders in previous studies (Kasama et al., 2005). Several studies with small samples showed that NLR was associated with major depression (MDD), and NLR significantly increased in patients with MDD compared with healthy controls (Demircan et al., 2016; Cai et al., 2017). A recently published study showed that NLR was associated with female depression in young and middle-aged Chinese adults (Meng et al., 2019). The aim of the present study was to investigate the relationship between NLR and geriatric depression among the elderly population in China.

## Materials and Methods

### Participants

Elderly adults who received medical examination at the Yinhang Community Health Service Center in Yangpu District, Shanghai from November 1 to December 30, 2017 were recruited in the study. The inclusion criteria for this study included: (1) age ≧ 60 years; (2) able to complete the Geriatric Depression Scale (GDS) independently. Exclusion criteria included: (1) with a history of neurological diseases that affect cognition or emotions, such as severe sequelae of cerebrovascular disease, craniocerebral injury, central nervous system infection; (2) taking antidepressant medication; (3) currently suffering from inflammatory diseases or with a history of autoimmune diseases; (4) with neuroendocrine neoplasm or hematologic disorders; (5) refused to participate in the study or with invalid GDS results. The study was approved by the Changhai Hospital Ethics Committee, and all participants signed informed consent. According to the inclusion and exclusion criteria, 665 participants were included in the final analysis, including 276 males (median age 68 years old) and 389 females (median age 67 years old).

### Methods

#### Collection of Demographic and Medical History Data

A standard questionnaire was used to collect demographic and medical history data of every participant. Marital (unmarried, married, divorced, or widowed) and smoking status (non-smoker, ex-smoker, or current smoking) were recorded. Hypertension was defined as systolic blood pressure greater than 140 mmHg or diastolic blood pressure greater than 90 mmHg or receiving antihypertensive therapy. Hyperlipidemia, Coronary heart disease (CHD), and cerebrovascular disease (CVD) were confirmed according to self-reported diagnosis.

#### Assessment of Depressive Symptoms

The depressive symptoms of participants were assessed with the simplified version of GDS, a self-report questionnaire consisting of 15 items (Liu et al., 1998). Participants were asked to give “yes” or “no” to each item, and the answer “yes” was to score one point. A total score was calculated and the participants with total score greater than five points were considered to be depressed. The reliability and validity of the questionnaire had been demonstrated in previous studies (Liu et al., 1998; Zhong et al., 2019).

#### Calculation of NLR

Fasting venous blood samples were obtained in the morning, then hematologic examination and blood biochemical test were accomplished. White blood cells (WBC), neutrophil, and lymphocyte counts and other hematologic and biochemical index were collected and NLR was calculated with the following formula: NLR, neutrophil count/lymphocyte count.

#### Statistical Analysis

The normally distributed continuous variables were described as mean ± standard deviation and *t* test was performed for statistical analysis. Non-normally distributed continuous variables were expressed as median (Q_25_, Q_75_) and Mann–Whitney test was performed for statistical analysis. The categorical variables were expressed as *n*% and Chi-square test was performed for statistical analysis. In order to estimate the association between NLR and depressive symptoms, NLR was converted to four levels according to quartiles in men and women, respectively, and logistic regression model was performed to explore the correlation between NLR levels and depressive symptoms. All the statistical tests were two-tailed and *P* < 0.05 were considered as statistically significant. All statistical analyses were performed by SPSS 24.0 software (IBM, Chicago, IL, United States).

## Results

### Comparison of Baseline Characteristics Between Different Genders

When we compared the baseline characteristics between men and women, several differences were found (Table 1). The average age of women was likely to be higher than that of men [67.0(63.0, 72.0) vs. 68.0(64.0, 72.0), *P* = 0.078). The prevalence of depressive symptoms were comparable between men and women. There were also differences in marital status between men and women (*P* = 0.033). Further more, there were more smokers or ex-smokers in men than women (*P* < 0.001). In contrast with men, women were more likely to suffer from hyperlipidemia (28.20 vs. 18.70%, *P* = 0.007), had higher level of total cholesterol (TC)(5.47 ± 0.53 vs. 5.03 ± 1.02, *P* < 0.001), low density lipoprotein cholesterin (LDL-C) (3.46 ± 0.96 vs. 3.22 ± 0.93, *P* = 0.002), higher density lipoprotein cholesterol (HDL-C) [1.57(1.33, 1.88) vs. 1.31(1.12, 1.60), *P* < 0.001], and had lower level of red blood cells (RBC) [4.61(4.39, 4.85) vs. 5.14(4.82, 5.40), *P* < 0.001], WBC [5.78(5.01, 6.59) vs. 6.22(5.33, 7.16), *P* < 0.001], neutrophil [3.18(2.66, 3.84) vs. 3.57(2.88, 4.31), *P* < 0.001], and thrombocyte (236.81 ± 54.49 vs. 225.10 ± 59.70, *P* < 0.003). There was a significant difference in NLR level between men and women [1.88(1.37, 2.44) vs. 1.63(1.28, 2.11), *P* = 0.01].

**TABLE 1:** Comparison of baseline characteristics between different genders.

**Characteristics**	**Men**	**Women**	***P* value**
No. of subjects	276	389	
Age (years)	68.0 (64.0, 72.0)	67.0 (63.0, 72.0)	0.078
Depressed	35.1%	34.4%	0.852
**Marital status (%)**			0.033
Unmarried	0.40%	0	
Married	96.10%	90.80%	
Divorced	0.90%	1.20%	
Widowed	2.60%	8.00%	
Hypertension (yes,%)	42.6%	43.5%	0.809
Hyperlipidemia (yes,%)	18.70%	28.20%	0.007
Diabetes (yes,%)	17.70%	16.20%	0.640
CHD (yes,%)	12.10%	15.2%	0.282
CVD (yes,%)	12.80%	10.40%	0.336
**Smoking status (%)**			<0.001
Non-smoker	50.20%	97.80%	
Smoker	23.10%	1.60%	
Ex-smoker	26.70%	0.50%	
TC (mmol/L)	5.03 ± 1.02	5.47 ± 0.53	<0.001
TG (mmol/L)	1.28 (0.93, 1.87)	1.38 (1.00, 1.88)	0.109
LDL-C (mmol/L)	3.22 ± 0.93	3.46 ± 0.96	0.002
HDL-C (mmol/L)	1.31 (1.12, 1.60)	1.57 (1.33, 1.88)	<0.001
FBG (mmol/L)	5.75 (5.23, 6.68)	5.63 (5.22, 6.32)	0.194
OGTT 2 h (mmol/L)	8.42 (6.39, 11.89)	8.66 (6.83, 11.35)	0.464
HbAlc (%)	5.90 (5.60, 6.30)	5.90 (5.60, 6.30)	0.947
RBC (×10^12^/L)	5.14 (4.82, 5.40)	4.61 (4.39, 4.85)	<0.001
RDW	12.40 (12.10, 12.90)	12.40 (12.00, 13.00)	0.103
WBC (×10^9^/L)	6.22 (5.33, 7.16)	5.78 (5.01, 6.59)	<0.001
Neutrophil (×10^9^/L)	3.57 (2.88, 4.31)	3.18 (2.66, 3.84)	<0.001
Lymphocyte (×10^9^/L)	1.93 (1.49, 2.37)	1.92 (1.63, 2.35)	0.694
Thrombocyte (×10^9^/L)	225.10 ± 59.70	236.81 ± 54.49	0.003
Monocyte (×10^9^/L)	0.46 (0.37, 0.55)	0.38 (0.31, 0.46)	<0.001
NLR	1.88 (1.37, 2.44)	1.63 (1.28, 2.11)	0.01

*CHD, coronary heart disease; CVD, cerebrovascular disease; TC, total cholesterol; TG, triglyceride; LDL-C, low density lipoprotein cholesterol; HDL-C, high-density lipoprotein cholesterol; FBG, fasting blood-glucose; OGTT, oral glucose tolerance test; HbAlc, glycosylated hemoglobin; RBC, red blood cell count; RDW, red blood cell distribution width; WBC, white blood cell count; NLR, neutrophil to lymphocyte ratio.*

### Differences Between Depressed and Normal Participants in Men

The present study showed that the prevalence of depressive symptoms was 35.1% in men. The differences between depressed and normal participants were presented in Table 2. Among male participants, the prevalence of depressive symptoms was higher for participants with hypertension than those without hypertension (51.10 vs. 38.10%, *P* = 0.040). Similarly, males with diabetes had a higher rate of depression than those without diabetes (23.8 vs. 14.4%), but the difference between the two groups did not reach statistical significance (*P* = 0.083). There was no significant difference in NLR levels between depressed men and normal controls [1.79(1.37, 2.48) vs. 1.92(1.40, 2.31), *P* = 0.714).

**TABLE 2:** Differences between depressed and normal participants in men.

**Characteristics**	**Normal**	**Depression**	***P* value**
No. of subjects	179	97	
GDS score	1 (0, 3)	6 (5, 8)	<0.001
Age (years)	68.5 (64.5, 72.5)	68 (64.0, 72.0)	0.541
**Marital status (%)**			0.480
Unmarried	0	1.20%	
Married	97.30%	94%	
Divorced	0.70%	1.20%	
Widowed	2.10%	3.60%	
Hypertension (yes,%)	38.10%	51.10%	0.040
Hyperlipidemia (yes,%)	18.20%	19.50%	0.802
Diabetes (yes,%)	14.70%	23.80%	0.083
CHD (yes,%)	12.20%	12.00%	0.973
CVD (yes,%)	11.90%	14.60%	0.538
**Smoking status (%)**			0.828
Non-smoker	51.50%	47.60%	
Smoker	26.30%	27.40%	
Ex-smoker	22.20%	25.00%	
TC (mmol/L)	5.05 ± 1.00	4.97 ± 1.10	0.598
TG (mmol/L)	1.27 (0.90, 1.89)	1.31 (0.96, 1.87)	0.634
LDL-C (mmol/L)	3.23 ± 0.91	3.18 ± 0.97	0.517
HDL-C (mmol/L)	1.33 (1.10, 1.60)	1.28 (1.13, 1.59)	0.712
FBG (mmol/L)	5.75 (5.20, 6.48)	5.78 (5.24, 7.35)	0.123
OGTT 2 h (mmol/L)	8.43 (6.81, 11.40)	8.13 (6.01, 12.69)	0.685
HbAlc (%)	5.90 (5.60, 6.30)	5.90 (5.55, 6.50)	0.781
RBC (×10^12^/L)	5.08 ± 0.49	5.16 ± 0.46	0.158
RDW	12.40 (12.10, 12.90)	12.40 (12.00, 13.00)	0.504
WBC (×10^9^/L)	6.17 (5.25, 7.04)	6.38 (5.43, 7.54)	0.167
Neutrophil (×10^9^/L)	3.47 (2.81, 4.25)	3.68 (3.02, 4.40)	0.151
Lymphocyte (×10^9^/L)	1.92 (1.49, 2.36)	1.96 (1.50, 2.39)	0.551
Thrombocyte (×10^9^/L)	225.02 ± 60.12	222.82 ± 59.03	0.882
Monocyte (×10^9^/L)	0.47 (0.37, 0.55)	0.45 (0.37, 0.53)	0.667
NLR	1.79 (1.37, 2.48)	1.92 (1.40, 2.31)	0.714

*GDS, Geriatric Depression Scale; CHD, coronary heart disease; CVD, cerebrovascular disease; TC, total cholesterol; TG, triglyceride; LDL-C, low density lipoprotein cholesterol; HDL-C, high-density lipoprotein cholesterol; FBG, fasting blood-glucose; OGTT, oral glucose tolerance test; HbAlc, glycosylated hemoglobin; RBC, red blood cell count; RDW, red blood cell distribution width; WBC, white blood cell count; NLR, neutrophil to lymphocyte ratio.*

### Differences Between Depressed and Normal Participants in Women

Compared with male participants, female participants had a similar morbidity of depression (34.4%). The differences between depressed and normal female participants were presented in Table 3. In female participants, those with depressive symptoms were more likely to report a history of hyperlipidemia or CVD than the normal ones (34.5 vs. 24.3%, *P* = 0.026 and 17.10 vs. 6.10%, *P* = 0.002, respectively). Compared with normal participants, neutrophils and NLR were significantly increased in female participants with depressive symptoms [3.15(2.61, 3.80) vs. 3.30(2.86, 3.98), *P* = 0.058 and 1.58(1.26, 2.06) vs. 1.74(1.43, 2.21), *P* = 0.020, respectively].

**TABLE 3:** Differences between depressed and normal participants in women.

**Characteristics**	**Normal**	**Depression**	***P* value**
No. of subjects	255	134	
GDS score	2 (1, 3)	6 (5, 8)	<0.001
Age (years)	67.0 (63.5, 72.5)	67.0 (64.0, 72.0)	0.336
**Marital status (%)**			0.082
Unmarried	0	0	
Married	88.70%	94.70%	
Divorced	0.90%	1.80%	
Widowed	10.40%	3.50%	
Hypertension (yes,%)	42.10%	46.20%	0.458
Hyperlipidemia (yes,%)	24.30%	35.50%	0.026
Diabetes (yes,%)	15.80%	17.10%	0.756
CHD (yes,%)	15.40%	15.00%	0.921
CVD (yes,%)	6.70%	17.10%	0.002
**Smoking status (%)**			0.430
Non-smoker	97.90%	97.60%	
Smoker	0.80%	0	
Ex-smoker	1.30%	2.40%	
TC (mmol/L)	5.49 ± 1.00	5.45 ± 1.03	0.734
TG (mmol/L)	1.37 (0.98, 1.86)	1.41 (1.03, 2.00)	0.263
LDL-C (mmol/L)	3.42 (2.84, 4.17)	3.35 (2.75, 4.04)	0.440
HDL-C (mmol/L)	1.58 (1.34, 1.84)	1.51 (1.32, 1.89)	0.197
FBG (mmol/L)	5.64 (5.23, 6.32)	5.59 (5.20, 6.30)	0.681
OGTT 2 h (mmol/L)	8.70 (6.85, 11.42)	8.38 (6.67, 11.05)	0.301
HbAlc (%)	5.90 (5.60, 6.30)	5.80 (5.60, 6.20)	0.534
RBC (×10^12^/L)	4.64 (4.38, 4.83)	4.60 (4.41, 4.90)	0.683
RDW	12.40 (12.00, 12.90)	12.30 (12.03, 12.88)	0.549
WBC (×10^9^/L)	5.77 (4.91, 6.54)	5.87 (5.14, 6.63)	0.199
Neutrophil (×10^9^/L)	3.15 (2.61, 3.80)	3.30 (2.86, 3.98)	0.058
Lymphocyte (×10^9^/L)	1.94 (1.65, 2.36)	1.90 (1.61, 2.30)	0.394
Thrombocyte (×10^9^/L)	229.00 (195.00, 273.00)	235.50 (206.75, 266.75)	0.726
Monocyte (×10^9^/L)	0.37 (0.31, 0.45)	0.39 (0.31, 0.49)	0.234
NLR	1.58 (1.26, 2.06)	1.74 (1.43, 2.21)	0.020

*GDS, Geriatric Depression Scale; CHD, coronary heart disease; CVD, cerebrovascular disease; TC, total cholesterol; TG, triglyceride; LDL-C, low density lipoprotein cholesterol; HDL-C, high-density lipoprotein cholesterol; FBG, fasting blood-glucose; OGTT, oral glucose tolerance test; HbAlc, glycosylated hemoglobin; RBC, red blood cell count; RDW, red blood cell distribution width; WBC, white blood cell count; NLR, neutrophil to lymphocyte ratio.*

### The Association Between NLR and Depression in Elderly Women

Subgroup analysis based on gender found that NLR was significantly increased in female participants with depressive symptoms. So, NLR was divided into four levels according to quartiles (Q_25_ = 1.32,Q_50_ = 1.71,Q_75_ = 2.28) in women, and then the classified NLR levels were included in logistic regression model and adjusted by hyperlipidemia and CVD. The results showed that NLR levels independently associated with depressive symptoms in elderly females, the odds ratios (ORs) of depression was 2.152(1.095, 4.227; *P* = 0.026) for the fourth compared with the first quartile of NLR (Table 4).

**TABLE 4:** The association between NLR and depression in elderly women.

	**β**	**95% CI**	***P* value**
Hyperlipidemia	0.612	0.368, 1.020	0.060
CVD	1.197	0.620, 2.313	0.592
**Levels of NLR**			
Level_1_(0.53–1.32)	–	–	–
Level_2_(1.33–1.71)	1.185	0.629, 2.332	0.600
Level_3_(1.73–2.28)	2.076	1.087, 3.963	0.027
Level_4_(2.28–5.80)	2.152	1.095, 4.227	0.026

*CVD, cerebrovascular disease; NLR, neutrophil to lymphocyte ratio.*

## Discussion

In this study we investigated the relationship between NLR and depressive symptoms in 665 elderly adults form Estern China. We found a comparable prevalence of depressive symptoms (GDS > 5) in females (34.4%) and males (35.1%). Depressive symptoms were associated to hypertension and diabetes in males, and to hyperlipidemia and CVD in females. Only in females NLR was significantly associated to depressive symptoms. To our best knowledge, the present study was the first to investigate the association between NLR and depressive symptoms among elderly adults in China.

As a simple and cost-effective bio-marker derived from routine hematology examination (Arbel et al., 2012), high levels of NLR are associated with increased oxidative stress and inflammatory cytokines (Kasama et al., 2005). Compared with normal controls, NLR was significantly higher in patients with MDD, and then reduced to the control group’s level after treatment with selective serotonin inhibitors (SSRIs), accompanied by remission of depressive symptoms (Demircan et al., 2016). In addition, multiple studies have shown the same association between increased NLR and depression (Demir et al., 2015; Aydin et al., 2016; Ekinci and Ekinci, 2017). A recently published study showed that NLR was associated with depression in female Chinese adults (aged 40.5 ± 11.6 years) (Meng et al., 2019). The present study showed that increased NLR was significantly related with depressive symptoms in elderly women, not men.

It is well known that gender is an important risk factor for mood disorders (Zhang et al., 2005), especially for depression. There are significant differences in clinical manifestations between male and female patients (Marcus et al., 2005). Thus, it can be inferred that the underlying pathogenesis of depression for men and women may be different. Considering that there was a significant difference in baseline NLR level between men and women, we performed a subgroup analysis of the relationship between NLR and depression in different genders, and the results showed that showed that NLR was independently associated with geriatric depression in women. Combined with the study of Meng, G, it can be confirmed that NLR is associated with depression among Chinese women (Meng et al., 2019). At the same time, we found that there were significant differences in baseline characteristics, especially medical history, between men and women. Different diseases may exert different effects on systemic inflammatory state. Therefore, the prevalence of each disease varies among men and women, which ultimately leads to a significant gender difference in the association between NLR and depression. Previous studies had indicated that postmenopausal women were more susceptible to depressive symptoms due to elevated inflammation, which maybe attributed to the decrease of estrogen (Straub, 2007). As mentioned above, the relationship between NLR and depression was consistent before and after menopause, suggesting that the role of estrogen in the etiology of depression is not well established.

Instead of recruiting participants from outpatient, the participants of this study were recruited from a randomly selected community to ensure the results more reliable. In addition, although the association between NLR and depression has been widely studied previously, the present study was the first to study it in the elderly. There were several limitations in this study. First, the cross-sectional design of the study precludes establishing causality of the association. Second, the diagnosis of depression is based on a self-rating scale rather than a professional clinical diagnosis. Third, incompletely including of social confounding variables may lead to bias in the results.

In summary, the current study showed that increased NLR levels were independently associated with geriatric depression in women. These findings suggested that inflammation may lead to female geriatric depression, and NLR can be used as a biomarker for geriatric depression in women.

## Data Availability Statement

The datasets generated for this manuscript are not publicly available. Requests to access the datasets should be directed to lmzsyd@163.com.

## Ethics Statement

The studies involving human participants were reviewed and approved by Changhai Hospital Ethics Committee. The patients/participants provided their written informed consent to participate in this study.

## Author Contributions

XB and CF conceived the trial design. BD, CF, HZ, XL, and CC collected the data. ML and BD analyzed the data and wrote the manuscript.

## Conflict of Interest

The authors declare that the research was conducted in the absence of any commercial or financial relationships that could be construed as a potential conflict of interest.
